# Supplementary material for: Phylogenetic and Evolutionary Comparison of Mitogenomes Reveal Adaptive Radiation of Lampriform Fishes
Source: Int J Mol Sci. 2023 May 15;24(10):8756. doi: 10.3390/ijms24108756 (PMC10218585; doi:10.3390/ijms24108756)
Supplement: Supplementary file 1 [file ijms-24-08756-s001.zip › ijms-2328803-supplementary.pdf]

Supplementary Tables and Figures

Supplementary Table S1 Species, GenBank accession number, and length of mitogenomes used in this study.

Species, Accession number, and length of mitogenomes used in this study.

	Order	Family	Species	Accession ID	Sequence length(bp)
1	Ateleopodiformes	Ateleopodidae	*Ateleopus purpureus*	AP012956.1	16653
2	Ateleopodiformes	Ateleopodidae	*Ijimaia dofleini*	NC_003179.1	16645
3	Argentiniformes	Opisthoproctidae	*Dolichopteryx minuscula*	AP012954.1	16875
4	Argentiniformes	Opisthoproctidae	*Bathylychnops exilis*	AP012953.1	16444
5	Argentiniformes	Bathylagidae	*Lipolagus ochotensis*	NC_004591.1	17338
6	Aulopiformes	Synodontidae	*Harpadon nehereus*	MH204885.1	16536
7	Aulopiformes	Synodontidae	*Saurida wanieso*	NC_025940.1	16552
8	Batrachoidiformes	Batrachoididae	*Porichthys myriaster*	AP006739.1	18910
9	Beryciformes	Berycidae	*Beryx splendens*	DQ996312.1	16529
10	Beryciformes	Berycidae	*Beryx mollis*	DQ993168.1	16537
11	Beryciformes	Cetomimidae	*Cetostoma regani*	NC_004389.1	16508
12	Carangiformes	Carangidae	*Seriola dumerili*	MZ398238.1	16530
13	Carangiformes	Carangidae	*Pseudocaranx dentex*	NC_058961.1	16569
14	Carangiformes	Carangidae	*Decapterus tabl*	MN399859.1	16545
15	Clupeiformes	Engraulidae	*Setipinna tenuifilis*	MT753060.1	16805
16	Clupeiformes	Clupeidae	*Pellonula vorax*	AP009231.1	16733
17	Clupeiformes	Clupeidae	*Etrumeus micropus*	AP009139.1	16979
18	Elopiformes	Elopidae	*Elops hawaiensis*	NC_005798.1	16713
19	Elopiformes	Megalopidae	*Megalops cyprinoides*	NC_005799.1	16412
20	Gadiformes	Gadidae	*Gadus chalcogrammus*	MW288643.1	16571
21	Gadiformes	Lotidae	*Lota lota*	KM201364.1	16547
22	Gobiiformes	Gobiidae	*Stiphodon percnopterygionus*	MW548257.1	16502
23	Gobiiformes	Gobiidae	*Sicyopus zosterophorus*	NC_058982.1	16471
24	Gobiiformes	Gobiidae	*Lentipes palawanirufus*	NC_058981.1	16497
25	Holocentriformes	Holocentridae	*Neoniphon sammara*	MZ329995.1	16473
26	Holocentriformes	Holocentridae	*Myripristis vittata*	MZ329989.1	16520
27	Carangiformes	Istiophoridae	*Istiophorus albicans*	AP006035.1	16514
28	Carangiformes	Istiophoridae	*Makaira mazara*	NC_012680.1	16534
29	Kurtiformes	Apogonidae	*Ostorhinchus fleurieu*	NC_056170.1	16521
30	Kurtiformes	Apogonidae	*Jaydia carinatus*	MN937193.1	16455
31	Beloniformes	Adrianichthyidae	*Oryzias latipes*	NC_004387	16714
32	Ophidiiformes	Ophidiidae	Bassozetus zenkevitchi	AP004405	16579
33	Lampriformes	Trachipteridae	*Trachipterus trachypterus*	NC_003166.1	16162
34	Lampriformes	Trachipteridae	*Zu cristatus*	NC_003167.1	15987
35	Lampriformes	Regalecidae	*Regalecus glesne*	AP012973.1	16781

36	Lampriformes	Veliferidae	*Metavelifer multiradiatus*	AP012972.1	16469
37	Lampriformes	Lophotidae	*Lophotus capellei*	AP012971.1	15814
38	Lampriformes	Lophotidae	*Eumecichthys fiski*	AP012970.1	16064
39	Lampriformes	Trachipteridae	*Desmodema polystictum*	AP012969.1	16121
40	Lampriformes	Lampridae	*Lampris guttatus*	NC_003165.1	15598
41	Lepisosteiformes	Lepisosteidae	*Lepisosteus oculatus*	NC_004744.1	16330
42	Myctophiformes	Myctophidae	*Notoscopelus elongatus kroyeri*	AP012262.1	16315
43	Myctophiformes	Myctophidae	*Bolinichthys pyrsobolus*	AP012261.1	15688
44	Ophidiiformes	Ophidiidae	*Neobythites unimaculatus*	AP018428.1	17794
45	Percopsiformes	Aphredoderidae	*Aphredoderus sayanus*	NC_004372.1	16717
46	Percopsiformes	Percopsidae	*Percopsis transmontana*	NC_003168.1	16601
47	Polymixiiformes	Polymixiidae	*Polymixia lowei*	NC_003181.1	16079
48	Salmoniformes	Salmonidae	*Salvelinus taranetzi*	MK695631.1	16473
49	Salmoniformes	Salmonidae	*Salvethymus svetovidovi*	MK695629.1	16481
50	Scombriformes	Nomeidae	*Cubiceps squamiceps*	MW401268.1	16654
51	Scombriformes	Trichiuridae	*Trichiurus haumela*	NC_042168.1	16655
52	Scombriformes	Scombridae	*Thunnus obesus*	KY400011.1	16624
53	Stylephoriformes	Stylephoridae	*Stylephorus chordatus*	NC_009948.1	16507
54	Syngnathiformes	Syngnathidae	*Hippocampus mohnikei*	KY798142.1	19855
55	Syngnathiformes	Syngnathidae	*Hippocampus hippocampus*	NC_045033.1	16524
56	Syngnathiformes	Syngnathidae	*Solegnathus hardwickii*	MH539788.1	15894
57	Trachichthyiformes	Monocentrida	*Monocentris japonicus*	NC_004392.1	16513
58	Zeiformes	Zeidae	*Zeus faber*	MT410863.1	16529
59	Zeiformes	Oreosomatidae	*Neocyttus rhomboidalis*	NC_004399.1	16519
60	Zeiformes	Oreosomatidae	*Allocyttus niger*	NC_004398.1	16595
61	Tetraodontiformes	Tetraodontidae	*Lagocephalus guentheri*	NC_059717.1	16567
62	Tetraodontiformes	Tetraodontidae	*Pao suvattii*	LC586271.1	16723
63	Cypriniformes	*Danionidae*	Danio rerio	NC_002333.2	16596
64	Esociformes	Esocidae	*Esox reichertii*	NC_023269	16909
65	Perciformes	Gasterosteidae	*Gasterosteus aculeatus*	NC_041244	16543

Supplementary Table S2 Annotation of three lampriform mitogenomes sequenced in this study. a, H: heavy strand.

Annotation of the *Lampris incognitus* mitogenome

Feature	Position	Nucleotide size (bp)	Start codon	Stop codon	Amino acid	Anticodon	Strand[a]
$tRNA^{Phe}$ (F)	1 -67	67				TTC	H
rrnS	68 -1026	959					H
$tRNA^{Val}$ (V)	1026-1096	71				GTA	H
rrnL	1097-2743	1647					H
trnL2	2744-2817	74				TTA	H
nd1	2818-3780	963	ATG	TAG	320		H
$tRNA^{Ile}$ (I)	3786-3855	70				ATC	H
tRNAGln (Q)	3857-3927	71				CAA	L
tRNAMet (M)	3927-3994	68				ATG	H
nd2	3995-5041	1047	ATG	TAG	348		H
tRNATrp (W)	5040-5109	70				TGA	H
tRNAAla (A)	5111-5179	69				GCA	L
tRNAAsn (N)	5180-5252	73				AAC	L
tRNACys (C)	5369-5432	64				TGC	L
tRNATyr (Y)	5433-5500	68				TAC	L
cox1	5502-7061	1560	GTG	AGG	519		H
RNASer(UCN) (S2)	7050-7120	71				TCA	L
tRNAAsp (D)	7123-7191	69				GAC	H
cox2	7202-7892	690	ATG	T	230		H
tRNALys (K)	7893-7964	72				AAA	H
atp8	7966-8133	168	ATG	TAA	55		H
atp6	8124-8807	684	ATG	TAA	227		H
cox3	8808-9591	784	ATG	T	261		H
tRNAGly (G)	9592-9660	69				GGA	H
nd3	9661-10010	351	ATG	TAG	116		H
tRNAArg (R)	10010-10078	69				CGA	H
nd4l	10078-10374	297	ATG	TAA	98		H
nd4	10368-11741	1374	ATG	TGA	457		H
tRNAHis (H)	11750-11818	69				CAC	H
RNASer(AGY) (S1)	11819-11885	67				AGC	H
RNALeu(CUN) (L1)	11893-11964	72				CTA	H
nd5	11965-13800	1836	ATG	TAA	611		H
nd6	13796-14317	522	ATG	AGG	173		L
tRNAGlu (E)	14319-14387	69				GAA	L
cytb	14390-15526	1137	ATG	TGA	378		H
tRNAThr (T)	15531-15602	72					
tRNAPro (P)							
Control region (CR)	15603-17376	1774					

Annotation of the *Trachipterus ishikawae* mitogenome

Feature	Position	Nucleotide size (bp)	Start codon	Stop codon	Amino acid	Anti-codon	Strand[a]
$tRNA^{Phe}$ (F)	1-67	67				TTC	H
rrnS	69-1013	946					H
$tRNA^{Val}$ (V)	1014-1086	73				GTA	H
rrnL	1088-2767	1680					H
trnL2	2768-2841	74				TTA	H
nd1	2842-3810	969	ATG	TAG	322		H
$tRNA^{Ile}$ (I)	3813-3881	69				ATC	H
tRNAGln (Q)	3881-3951	71				CAA	L
tRNAMet (M)	3951-4019	69				ATG	H
nd2	4020-5066	1047	ATG	TAG	348		H
tRNATrp (W)	5065-5137	73				TGA	H
tRNAAla (A)	5262-5330	69				GCA	L
tRNAAsn (N)	5331-5404	74				AAC	L
tRNACys (C)	5408-5471	64				TGC	L
tRNATyr (Y)	5472-5539	68				TAC	L
cox1	5541-7091	1551	GTG	TAA	516		H
RNASer(UCN) (S2)	7092-7162	71				TCA	L
tRNAAsp (D)	7166-7233	68				GAC	H
cox2	7248-7938	705	ATG	T	230		H
tRNALys (K)	7939- 8012	74				AAA	H
atp8	8014-8181	168	ATG	TAA	55		H
atp6	8172- 8855	684	ATG	TAA	227		H
cox3	8855- 9638	784	ATG	T	261		H
tRNAGly (G)	9639- 9707	69				GGA	H
nd3	9708- 10058	351	ATG	TAG	116		H
tRNAArg (R)	10057- 10125	69				CGA	H
nd4l	10126- 10422	297	ATG	TAA	98		H
nd4	10416- 11816	1401	ATG	T	466		H
tRNAHis (H)	11797- 11866	70				CAC	H
RNASer(AGY) (S1)	11867- 11933	67				AGC	H
RNALeu(CUN) (L1)	11937- 12009	73				CTA	H
nd5	12010- 13842	1833	ATG	T	610		H
nd6	13835 -14359	525	ATG	TGA	174		L
tRNAGlu (E)	14360- 14428	69				GAA	L
cytb	14433 -15569	1137	ATG	TGA	378		H
tRNAThr (T)	15574- 15644	71				ACA	H
tRNAPro (P)	15644- 15713	70				CCA	L
Control region (CR)	15714-18548	2835					

Annotation of the *Regalecus russelii* mitogenome

Feature	Position	Nucleotide size (bp)	Start codon	Stop codon	Amino acid	Anticodon	Strand[a]
tRNA^{Phe} (F)	1-67	67				TTC	H
rrnS	68-1014	947					H
tRNA^{Val} (V)	1015-1087	73				GTA	H
rrnL	1089-2757	1669					H
trnL2	2758-2831	74				TTA	H
nd1	2832-3800	969	ATG	TAG	322		H
tRNA^{Ile} (I)	3803-3872	70				ATC	H
tRNAGln (Q)	3872-3942	71				CAA	L
tRNAMet (M)	3942-4010	69				ATG	H
nd2	4011-5057	1047	ATG	TAA	348		H
tRNATrp (W)	5057-5129	73				TGA	H
tRNAAla (A)	5301-5369	69				GCA	L
tRNAAsn (N)	5372-5444	73				AAC	L
tRNACys (C)	5445-5509	65				TGC	L
tRNATyr (Y)	5510-5577	68				TAC	L
cox1	5579-7129	1551	GTG	TAA	516		H
RNASer(UCN) (S2)	7130-7200	71				TCA	L
tRNAAsp (D)	7204-7272	69				GAC	H
cox2	7287-7977	705	ATG	T	230		H
tRNALys (K)	7978-8051	74				AAA	H
atp8	8053-8220	168	ATG	TAA	55		H
atp6	8211-8894	684	ATG	TAA	227		H
cox3	8894-9677	784	ATG	T	261		H
tRNAGly (G)	9678-9746	69				GGA	H
nd3	9747-10097	351	ATG	TAG	116		H
tRNAArg (R)	10096-10164	69				CGA	H
nd4l	10165-10461	297	ATG	TAA	98		H
nd4	10455-11828	1374	ATG	TGA	457		H
tRNAHis (H)	11836-11904	69				CAC	H
RNASer(AGY) (S1)	11905-11973	69				AGC	H
RNALeu(CUN) (L1)	11977-12048	72				CTA	H
nd5	12049-13881	1833	ATG	TAA	610		H
nd6	13871-14395	525	ATG	TGA	174		L
tRNAGlu (E)	14396-14464	69				GAA	L
cytb	14467-15603	1137	ATG	TGA	378		H
tRNAThr (T)	15608-15678	71				ACA	H
tRNAPro (P)	15678-15746	69				CCA	L
Control region (CR)	15747-16538	792					

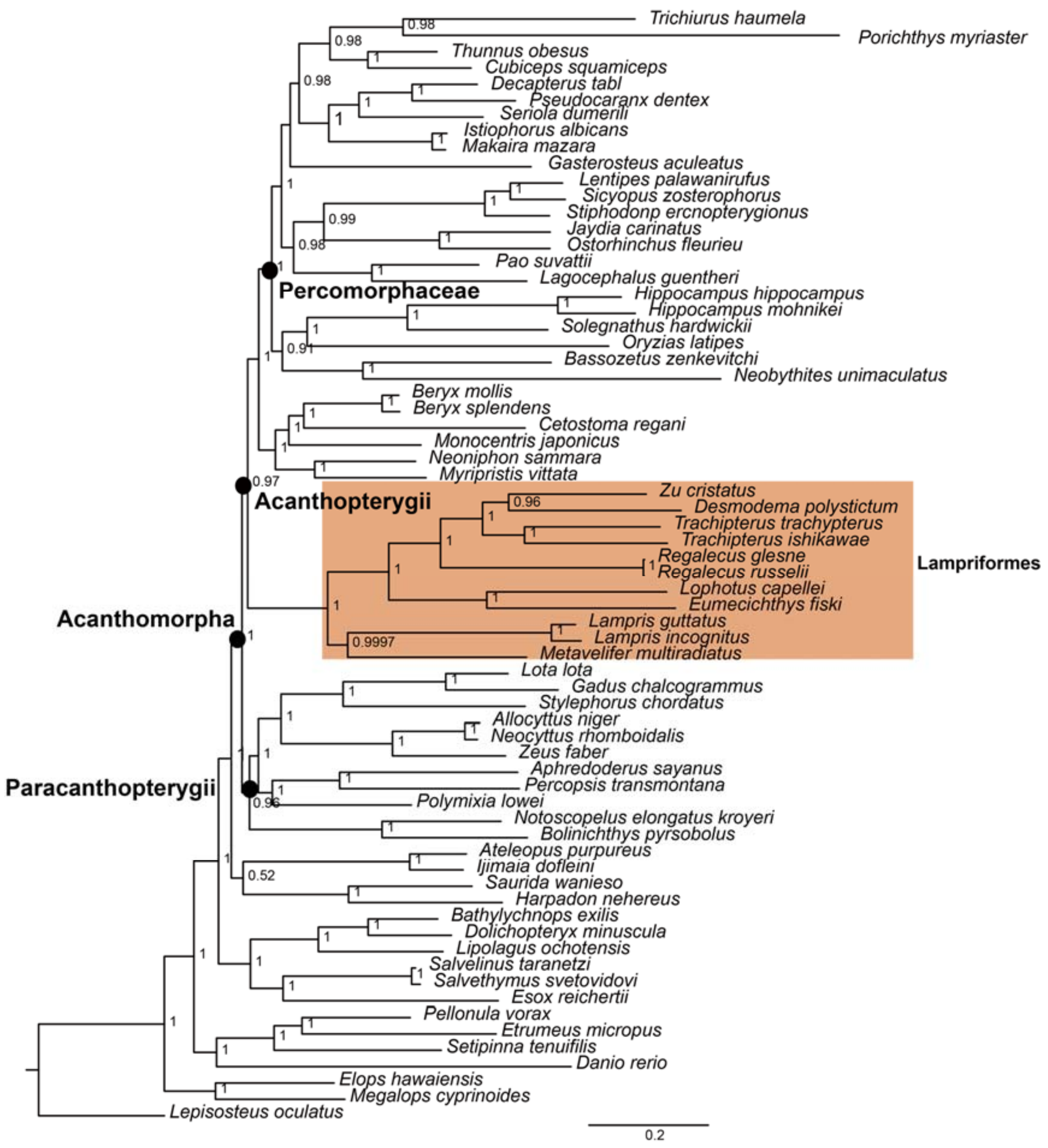

Supplementary Figure S1. Phylogenetic tree constructed by MrBayes methods based on 13 PCGs of 68 species from teleost fishes mitogenomes. *L. oculatus* was chosen as outgroup. Node numbers represent Bootstrap support (BS) values.

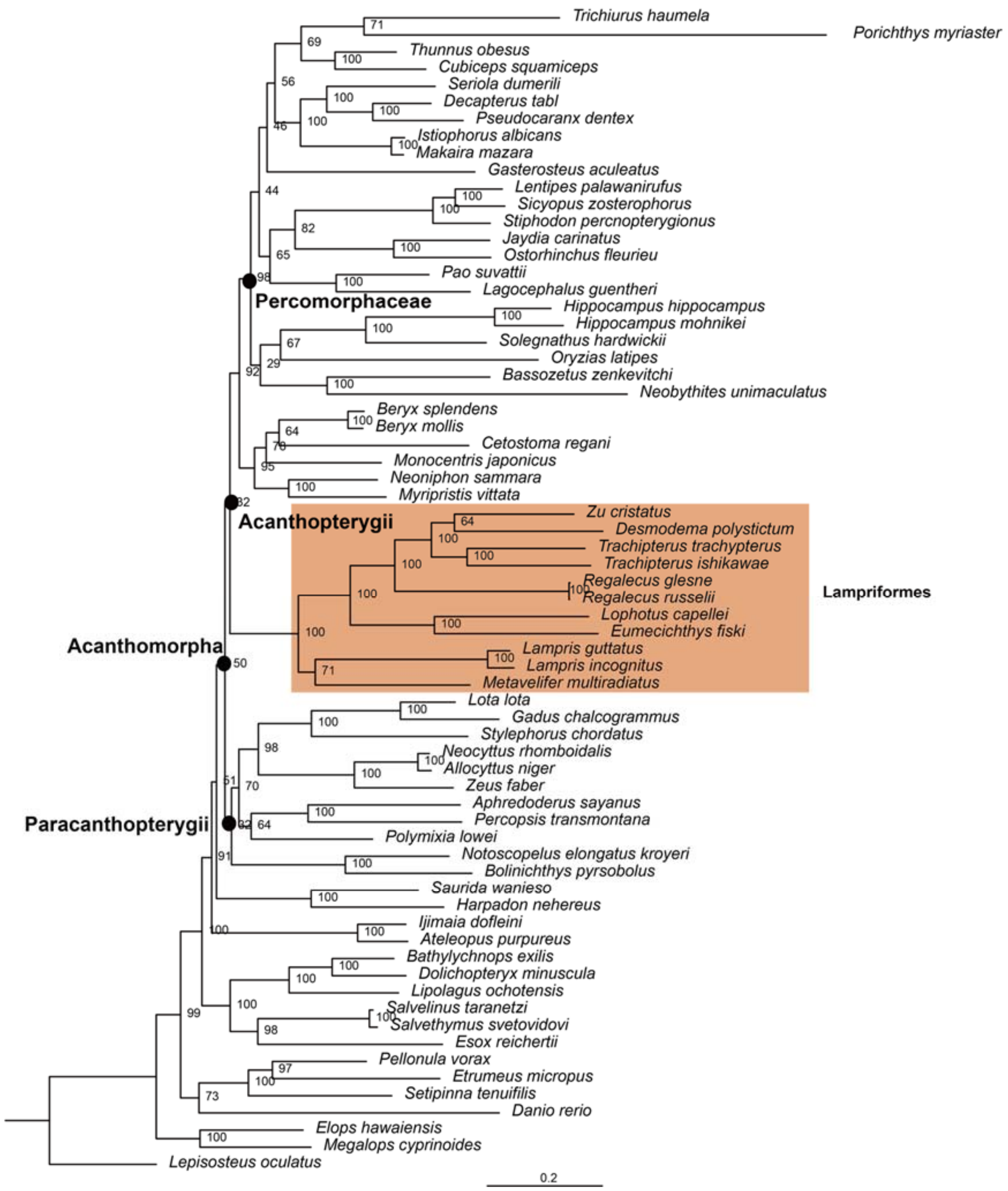

Supplementary Figure S2. Phylogenetic tree constructed by ML methods based on 13 PCGs of 68 species from teleost fishes mitogenomes. *L. oculatus* was chosen as outgroup. Node numbers represent Bootstrap support (BS) values.

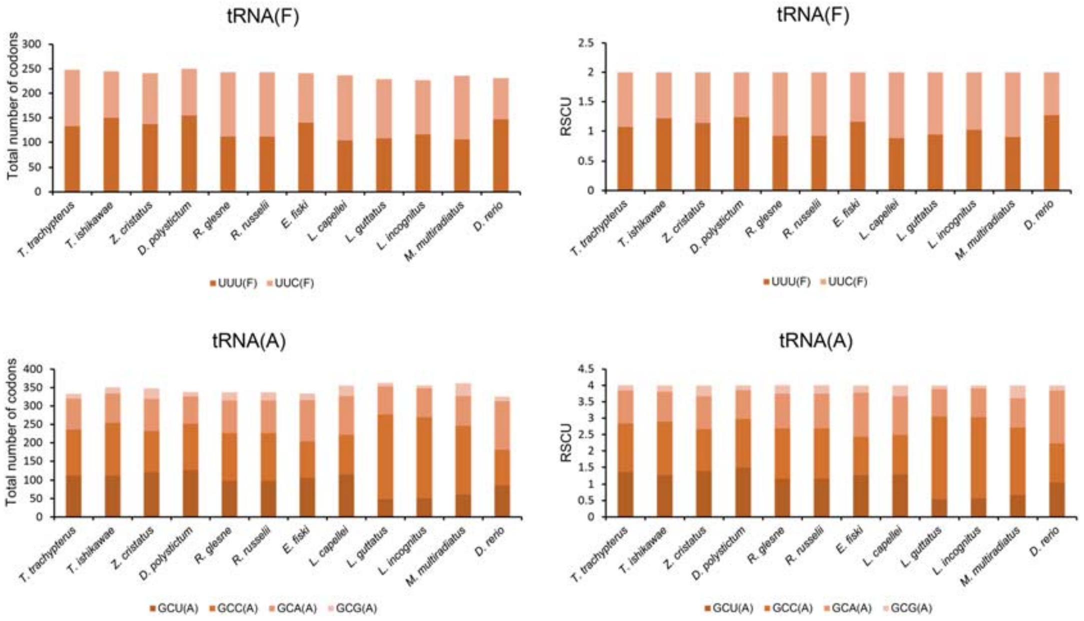

Supplementary Figure S3. Relative synonymous codon usage (RSCU) of the mitogenomes of Lampriformes.

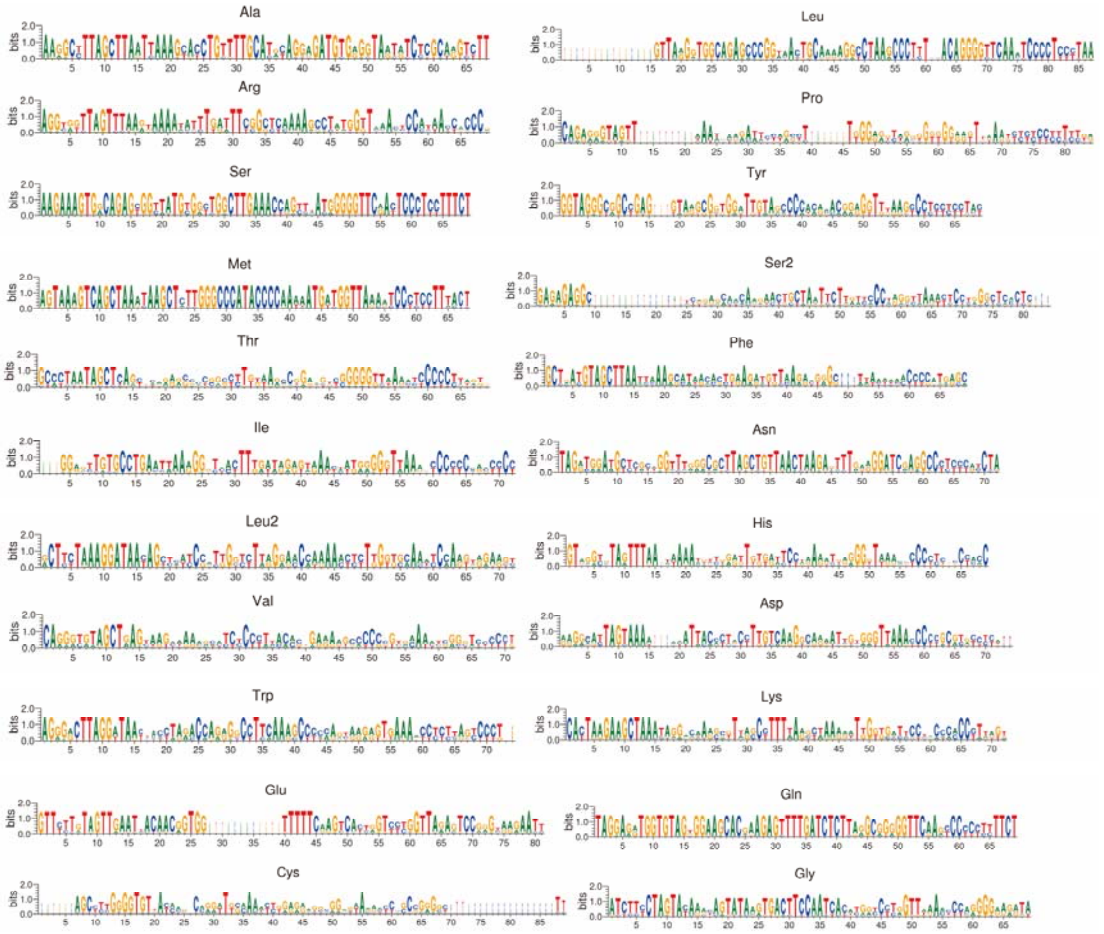

Supplementary Figure S4. Sequence logos of 22 tRNAs of lampriform mitogenomes.
